# Development of shelf-stable reagents and assay kits for bioluminescence applications using the capillary-assisted vitrification platform stabilization technology

**DOI:** 10.1371/journal.pone.0354533

**Published:** 2026-11-05

**Authors:** Shari Radford, Yolanda Peris-Taverner, Michael Dibble, Kevin Corn, Tian Zhu, Shannon Martello, McKenzie Mayeaux, Amanda Ladd, Sankar Renu, Tejasvi Chunduri, Aniket Jadhav, Melanie Dart, Marjan Rafat, Mary Shank-Retzlaff, Laura L. Bronsart

**Affiliations:** 1 Ambient Biosciences, Ann Arbor, Michigan, United States of America; 2 Promega Corp., Madison, Wisconsin, United States of America; 3 Department of Chemical and Biomolecular Engineering, Vanderbilt University, Nashville, Tennessee, United States of America; 4 Department of Biomedical Engineering, Vanderbilt University, Nashville, Tennessee, United States of America; 5 Department of Radiation Oncology, Vanderbilt University Medical Center, Nashville, Tennessee, United States of America; Anhui Polytechnic University, CHINA

## Abstract

Luminescence is a powerful method for detecting trace analytes and monitoring biological processes. However, most bioluminescence reagents, including luciferase and its substrates, are sensitive to temperature, limiting their usable shelf lives, and resulting in inconsistent performance. Enhancing the stability of these reagents could reduce signal variability between experiments, simplify workflows, and address cold chain storage issues. In this study, we demonstrate the application of the platform stabilization technology, capillary-assisted vitrification (CAV), as a tool to stabilize different luciferases and their substrates, and the application of the stabilized reagents in both *in vitro* and *in vivo* bioluminescent assays. We demonstrate that CAV-stabilized reagents can be stored and shipped ambiently, maintain consistent performance over time, and are suitable for use in cell viability quantification, tumor monitoring, *in vivo* imaging, microbial detection, and immunoassays. Additionally, different reagents can be co-formulated to make ready-to-use assay kits that can also be shipped and stored ambiently. Our results demonstrate that CAV stabilization is a viable alternative to traditional storage methods, with broad potential to improve bioluminescence workflows.

## Introduction

Luminescence has emerged as a powerful tool for biological and biochemical research [1–5]. Luminescence is defined as the production and emission of visible light resulting from a chemical reaction that releases energy in the form of light instead of heat. The term bioluminescence describes luminescent reactions that occur in nature and are typically the result of an enzymatic reaction. The enzyme reacts with a small molecule substrate in the presence of specific co-factors which may include oxygen, adenosine triphosphate (ATP) and calcium or other metals. Bioluminescence-based imaging has been widely adopted as a tool for identifying and monitoring tumor growth and monitoring viral infection in animal models [6–8]. Bioluminescence can also be used to assess the efficacy and toxicity of drug candidates, evaluate cell viability, and detect microbial contamination in hospital and food production settings [9–12].

Despite the broad utility of bioluminescence-based assays, the limited thermal stability of the enzymes and substrates used to generate the assay signals continues to present logistical challenges [13]. The reagents require long-term storage at ultra-cold temperatures and the performance of the reagents can degrade over time, adversely impacting sensitivity and assay reproducibility and limiting their use in point-of-care applications. Although significant effort has been made to enhance the stability of bioluminescent reagents through genetic engineering, chemical modification and specialized formulations [14–20], the components remain highly temperature dependent.

Techniques such as lyophilization, and the more modern lyobead technology, have been successfully used to enhance stability of a broad range of reagents and samples and have occasionally led to improved assay performance [21–23]. However, these processes can be both cost prohibitive and damaging to sensitive biomolecules. Lyophilization exposes biomolecules to a range of chemical and physical stresses including cold denaturation, cryoconcentration, pH shifts, unfavorable protein-excipient interactions, and exposure to solid-liquid interfaces as ice crystals form [24–26]. Together, these stresses can compromise biomolecule integrity, reduce functional activity, and ultimately lead to inconsistent performance.

Luminescent substrates present an additional stabilization challenge. Most luminescent substrates are hydrophobic and may exhibit limited solubility making them challenging to lyophilize on their own and particularly difficult to lyophilize when incorporated into a mixture. Lyophilization of highly hydrophobic materials generally requires extensive, molecule-specific optimization, and may or may not produce satisfactory results [27].

Novel stabilization technologies that can be applied by researchers to stabilize diverse bioluminescent reagents are therefore still needed. The ideal solution should be easy to deploy in a laboratory setting, require minimal capital investment, require minimal molecule-specific optimization and facilitate production of assay master mixes and kits.

We previously described the development of a novel stabilization technology called Capillary-Assisted Vitrification (CAV, referred to as Capillary Mediated Vitrification or CMV in previously published work) [28–31]. Like lyophilization, CAV achieves stabilization by transitioning sensitive biomolecules into an amorphous glass through a process referred to as vitrification. In the glassy state, both intra- and inter-molecular motion is restricted preventing collisions and the molecular conformational changes required for many common degradation pathways [32]. Stability is further achieved by removing bulk water and thereby slowing or halting water-mediated degradation processes such as deamidation.

Compared to lyophilization, CAV is a relatively gentle process and does not require a freezing step. In the CAV process, the reagent of interest is mixed with a pre-formulated stabilization buffer, referred to as BioFix™ or BioFix+™, and applied to a solid, porous material referred to as a scaffold. The formulated liquid embeds into the pores of the scaffold due to capillary action. The reagent-loaded scaffold is then placed into a purpose-built vacuum chamber, referred to as the Ambient Stabilizer, and dried under coarse vacuum. A combination of the high surface area provided by the capillary network within the scaffold and a controlled evacuation of the vacuum chamber allows for gentle desiccation of the sample matrix avoiding both boiling and freezing the liquid.

Although the CAV technology has been applied successfully for the stabilization of antibody reagents and PCR master mixes [30,31], only limited work has been done using bioluminescent reagents. In a previous study, we demonstrated that stabilized luciferase could be stored for up to 6 weeks at room temperature with minimal impact on the enzyme activity but did not evaluate long-term stability or the suitability of the stabilized material in real-world analytical methods [28]. Here, we expand on our previous work by demonstrating that the CAV technology can be applied not only to luciferase, but also to luciferin and furimazine, two commonly used bioluminescent substrates. We demonstrate long-term stability of luciferase, luciferin, and furimazine and evaluate batch-to-batch consistency for stabilized luciferase. We establish that the reagents can be co-formulated into a variety of ready-to-use assay master mixes using a common stabilization protocol. Finally, we evaluate the suitability of the CAV stabilized reagents in a wide range of *in vitro*, *in vivo,* and biochemical methods including cell viability assays, tumor imaging, and mix-and-read bioluminescent immunoassays. Overall, the results of this investigation indicate that CAV-stabilization is an attractive platform stabilization technology for stabilizing bioluminescent reagents.

## Results

### Performance and stability of luciferase and luciferin

Implementation of bioluminescence applications under real-world conditions requires the ability to store reagents for periods of 6 months or more and to withstand short-term exposures to extreme temperature resulting from temperature excursions during shipping or storage. Thus, we initiated long-term stability studies of luciferase and luciferin at room temperature and a short-term stability study at 55 °C. At each time point, the activity of the CAV-stabilized reagents was evaluated by comparing the performance of the CAV-stabilized luciferase or luciferin to a control preparation made from reagents that were stored frozen and subsequently thawed and prepared on the day of use (Fig 1).

**Fig 1:**
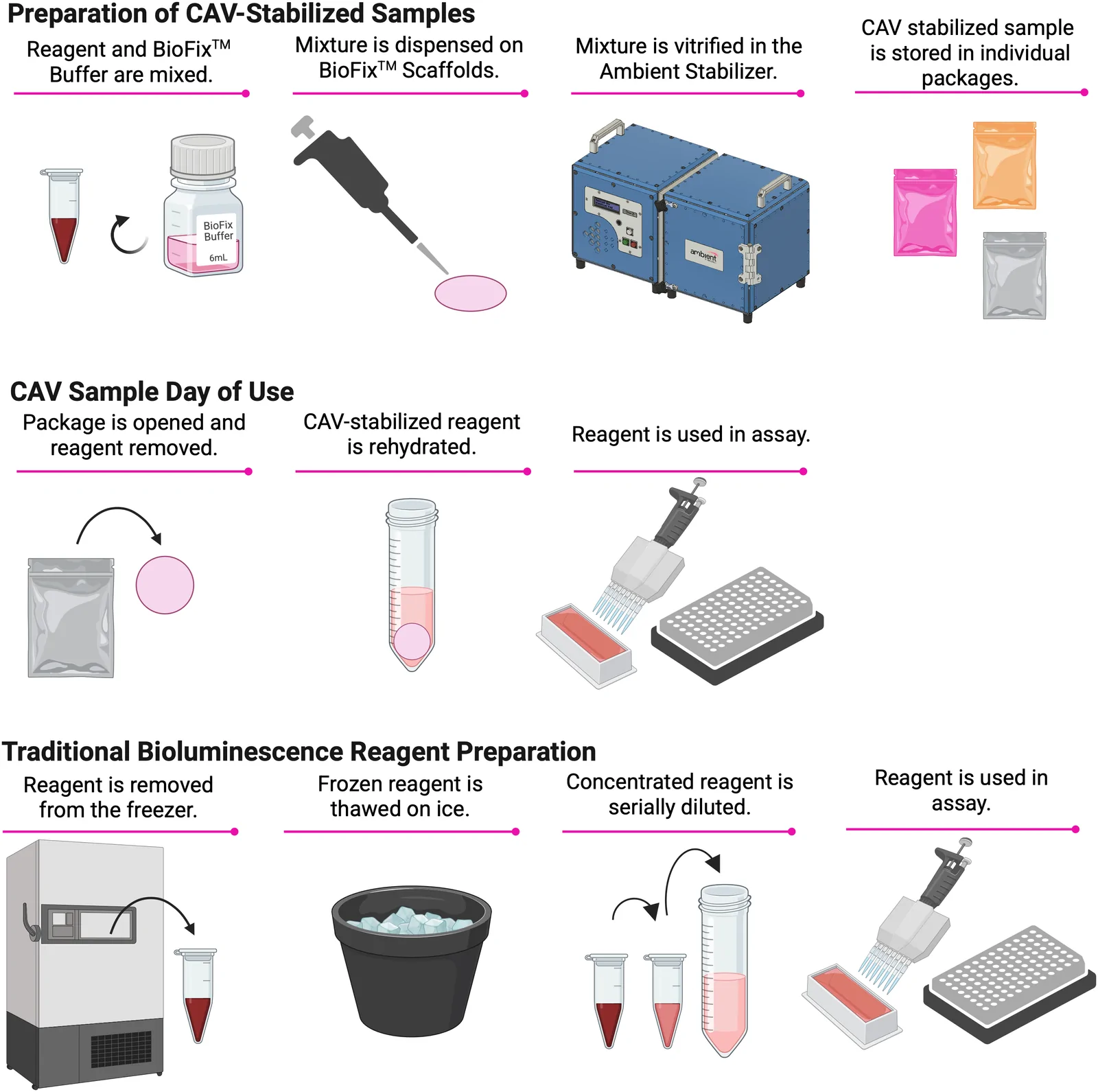
Schematic diagram of the CAV-stabilization process (top), rehydration and day-of-use protocol for CAV-stabilized samples (middle), and the procedure used to prepare traditional bioluminescent reagents and liquid controls from frozen reagent stocks (bottom). Reprinted from Bronsart, L. 2026 https://BioRender.com/k45a805, under a CC BY license, with permission from Biordender original copyright 2026.

At the start of the stability study, we first evaluated the recovery of the two reagents immediately post-stabilization. As shown in Fig 2A, the signals obtained for the CAV-stabilized luciferin were comparable to the control preparation. The dose response curves generated using the CAV-stabilized and control reagents were evaluated graphically and analyzed using a two-sided t-test; the results were not statistically different between the stabilized and control material (p-value = 0.31). When the results for the CAV-stabilized luciferase were compared to the control preparation of luciferase (Fig 2B), a statistically significant difference between CAV-stabilized and the control preparation of luciferase was observed (p-value = 0.009). The CAV-stabilized material provided slightly higher signals and lower variability across replicate results compared to the frozen control. Despite the small difference in signal, the data indicates that the stabilization buffer does not interfere with the enzymatic reaction that produces the luminescent signal and that the process results in little or no loss of material.

**Fig 2:**
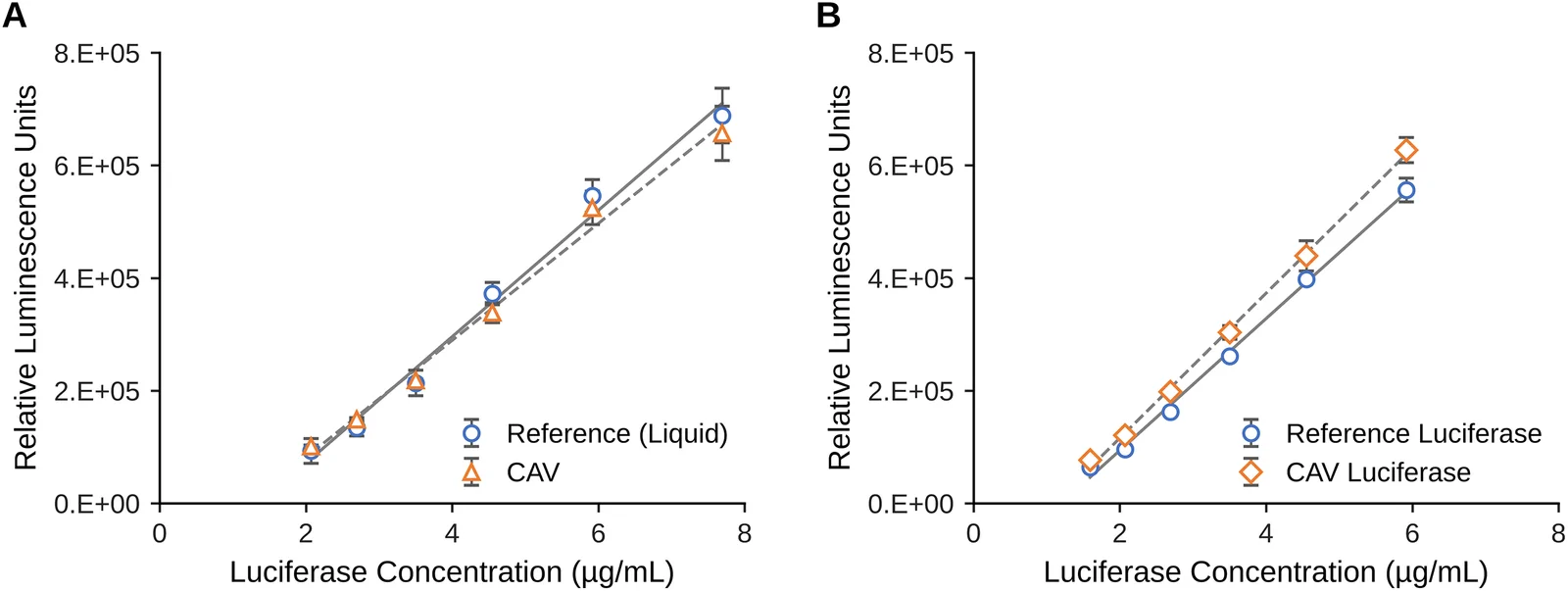
(A) Comparison of luminescent signal generated using CAV-stabilized luciferin (orange triangles) compared to the control material prepared from freshly thawed luciferin (blue circles). (**B**) Comparison of luminescent signal generated using CAV-stabilized luciferase (orange diamonds) compared to the control material prepared from freshly thawed luciferase (blue circles). Error bars show standard deviation across the replicate results (n = 3).

We further evaluated the performance of the CAV-stabilized reagent by calculating the assay limit of quantitation (LOQ) for stabilized reagents and control preparations. Across experiments, the estimated LOQ ranged from 0.7-2.5 µg/mL using control preparations of luciferin and luciferase. Using CAV-stabilized luciferin or luciferase resulted in an LOQ of 2.3 and 1.1 µg/mL, respectively. This is well within the range observed for the reference preparations. In all cases, the dose response curves exhibited coefficient of determination (R^2^) of >0.99. Thus, we conclude that the stabilization process had no adverse impact on linearity or sensitivity.

Next, aliquots of CAV-stabilized reagents and controls were placed on stability. At each time point, the activity of the CAV-stabilized reagent and the liquid controls were again compared to a reference standard prepared from freshly thawed luciferase and luciferin. At the end of the study, stability data were evaluated graphically to estimate the degradation rates at each temperature. As shown in S1 Fig and S2 Fig, the CAV-stabilized materials show a linear trend when the natural log of activity is plotted versus time, indicating the degradation process can be described by a first-order reaction rate. According to the differential rate law, the slope of this line is equal to -k, where k is the rate constant. The half-life (t_1/2_) can then be calculated using the equation:


$$\begin{array}{c} t_{\frac{\text{1}}{\text{2}}}=\frac{\text{ln}\text{2}}{k} \end{array}$$

The t_1/2_ values are shown in Table 1. The t_1/2_ for the CAV-stabilized luciferin was estimated to be 39 months at room temperature and 2.1 months at 55 °C. The t_1/2_ value for CAV-stabilized luciferase was estimated to be 19 months at 55 °C. Although a linear trend was observed for luciferase stored in the CAV-form at 25 °C, the half-life could not be accurately measured. The activity of the CAV-stabilized luciferase was essentially flat across the nearly 2 year study, showing little or no downward trend and resulting in a correlation coefficient of 0.13. Even after 22 months of storage, the enzyme maintained 97% activity (S2 Fig). Based on this data, we conclude that the t_1/2_ will significantly exceed 2 years at room temperature.

**Table 1:** Estimated half-lives (t_1/2_) for luciferin and luciferase stored in liquid or post-CAV-stabilization at various temperatures.

Reagent	Storage Temp. (°C)	Liquid Formulation	CAV Stabilized
		t_1/2_ (months) [95% CI]	
Luciferin	25	0.1 [0.07, 0.4]	39 [34, 46]
	55	<1 day^a^	2.1 [1.8, 2.4]
Luciferase	25	Highly variable activity at all timepoints	>2 years^b^
	55	<1 day^a^	19 [14, 28]

^a^No activity was detected after 1 day at 55 °C; t_1/2_ could not be calculated and is therefore reported as <1 day.

^b^Due to small degree of change in luciferase activity during the study, the correlation between the natural log of activity and time is poor (R^2^ = 0.13) and the reaction rate and t_1/2_ cannot be accurately determined for this condition.

It is important to note that stability was assessed based on activity within the target assay only. No structural testing was performed. While activity is typically correlated with structure and is considered a key indicator of reagent stability, further testing is required to determine whether any structural changes occurred during storage.

Except for luciferin stored at room temperature (t_1/2_ = 0.1 months), we were unable to determine t_1/2_ for the liquid formulations. When stored as a liquid at 55 °C, both luciferase and luciferin lost all activity in less than 1 day. Test results for the luciferase formulation stored as a liquid at 25 °C were not reproducible between time points or between repeated experiments. Due to this variability, the performance of the liquid luciferase stored under this condition is considered unacceptable for an analytical reagent.

Overall, our stability evaluation confirms the following: 1) the CAV process significantly enhances the stability of both luciferase and luciferin, 2) the CAV-stabilized reagents can be stored for greater than 6 months at room temperature, and 3) the CAV-stabilized reagents can withstand short-term temperature excursions of up to 55 °C. Together, the data suggests that the CAV-stabilized reagents are suitable for ambient distribution and storage.

To further evaluate the performance of the CAV-luciferase with respect to reagent consistency over time and between stabilization cycles, a scale-up study was performed. In each run, a total of 512 scaffolds were prepared and stabilized in a single stabilization cycle. A minimum of 6 scaffolds were tested immediately post-stabilization and again after the scaffolds had been stored for 9 months at room temperature. The mean activity across all four batches was 103% both post-stabilization and after 9 months of storage (Table 2). The relative standard deviation (RSD) across scaffolds produced within a batch ranged from 1.9% to 7.8%. The RSD across the batches was 7.2% at time 0 and 5.1% after 9 months of storage. This data demonstrates that the CAV-process is highly consistent and further confirms the observation that the stabilized reagents are stable for months or years at room temperature.

**Table 2:** Evaluation of CAV process consistency across four separate stabilization cycles. Comparison of luciferase activity on scaffolds immediately post-stabilization and after 9 months of storage at room temperature.

Full-Scale Run	Post Stabilization	9M, RT
	% Luciferase Activity (RSD)	
Batch 1	99 (1.9%)	97 (4.9%)
Batch 2	95 (2.8%)	101 (3.4%)
Batch 3	112 (6.2%)	107 (4.0%)
Batch 4	105 (7.8%)	108 (5.8%)
Average	103 (7.2%)	103 (5.1%)

### Experimental applications

#### Suitability for imaging and reporter gene assays.

We next evaluated the suitability of the CAV-stabilized luciferin for use in an *in vivo* imaging application and compared results to those generated with luciferin that was stored lyophilized or frozen. We quantified tumor burden using a 4T1 mouse mammary carcinoma model. Tumor size was monitored for up to 21 days after 4T1 tumor inoculation (Fig 3), and the results obtained with bioluminescence imaging were compared to measurements made with calipers.

We observed no statistical difference in the relationship between caliper measurements and bioluminescence signal of tumor growth between the luciferin groups (Fig 3A–C). Although the CAV-stabilized luciferin experienced temperatures exceeding 41 °C during shipment as measured using a temperature tracker, we found that both *in vivo* bioluminescence on day 16 post-inoculation and *ex vivo* tumor measurements were comparable in mean signal and the CAV-stabilized luciferin exhibited a smaller variation in signal compared to the frozen control luciferin (Fig 3C-D). This side-by-side analysis suggests that the stabilization process results in a functional and stable product equivalent to the current gold standard in bioluminescence imaging of tumors in mice.

**Fig 3:**
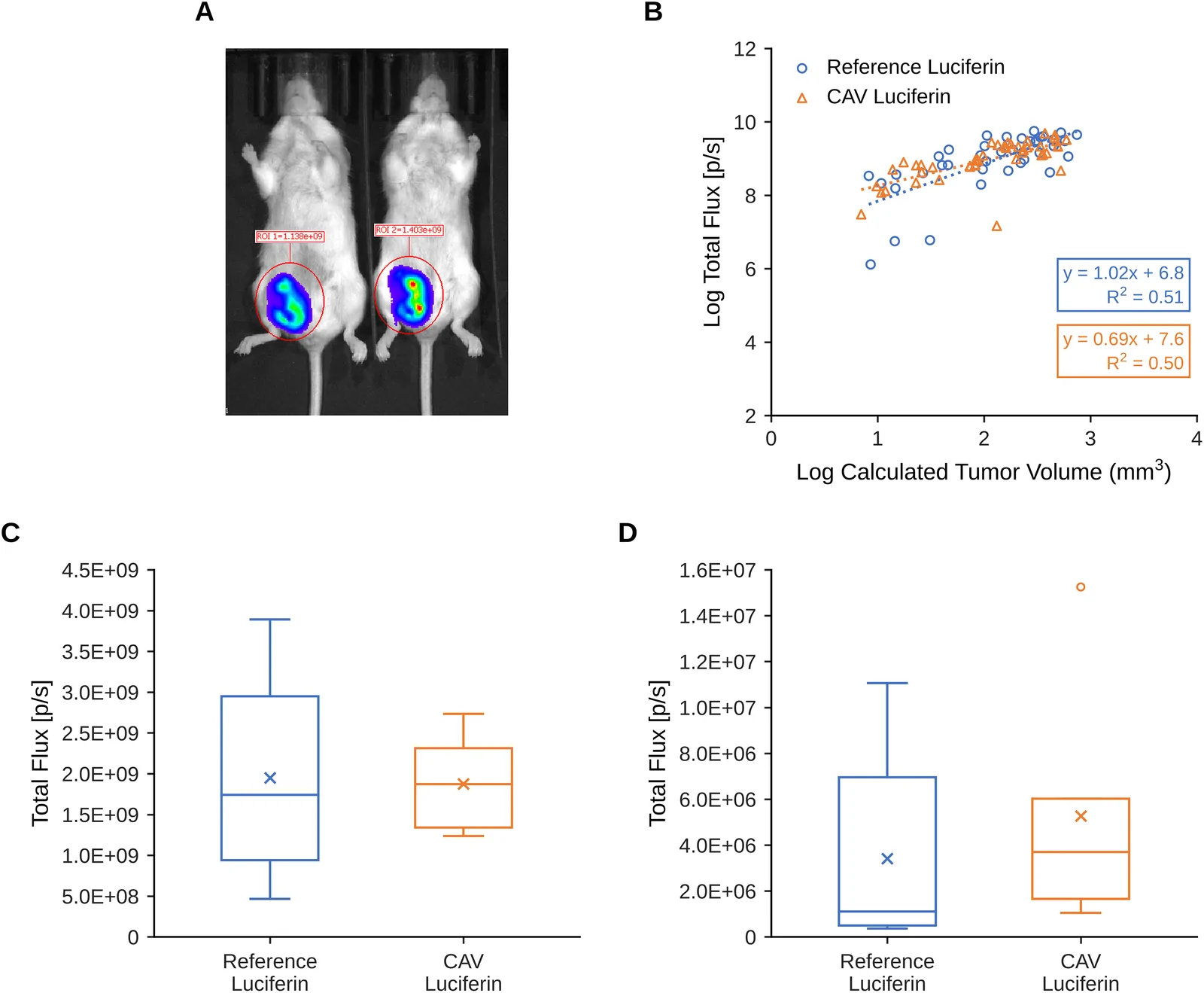
CAV-stabilized luciferin for quantitation of tumor burden and *in vivo* imaging. *In vivo* tumor imaging reveals consistent bioluminescence measurements between frozen reference (blue) or CAV-stabilized (orange) luciferin. (**A**) Mice implanted with 4T1 breast cancer cells in the #4 right mammary fat pad and imaged with the frozen reference luciferin (2 of 15 mice shown) (left) or CAV-stabilized luciferin (right). (**B**) Relationship between bioluminescence intensity and tumor volume based on caliper measurements. (**C**) Comparison of *in vivo* bioluminescence on day 16 post-inoculation (n = 7 reference, n = 8 CAV-stabilized). (**D**) Comparison of *ex vivo* tumor bioluminescence (n = 7 per group). The boxplots in C and D show the mean (x), median, the interquartile range (IQR), and ± 1.5 x IQR.

Bioluminescence is also frequently used to monitor the expression of reporter genes in cell culture applications. Cells containing the reporter gene are cultured, subjected to the test condition, and treated with luciferin or another substrate to produce a luminescent signal that is proportional to the level of reporter gene expression. To assess whether vitrified luciferin could be used in this type of application, luciferase-expressing 4T1 mouse breast cancer cells were prepared as a suspension culture and then serially diluted. Fig 4 shows that the frozen and CAV-vitrified luciferin signal are not statistically different based on ANCOVA results.

**Fig 4:**
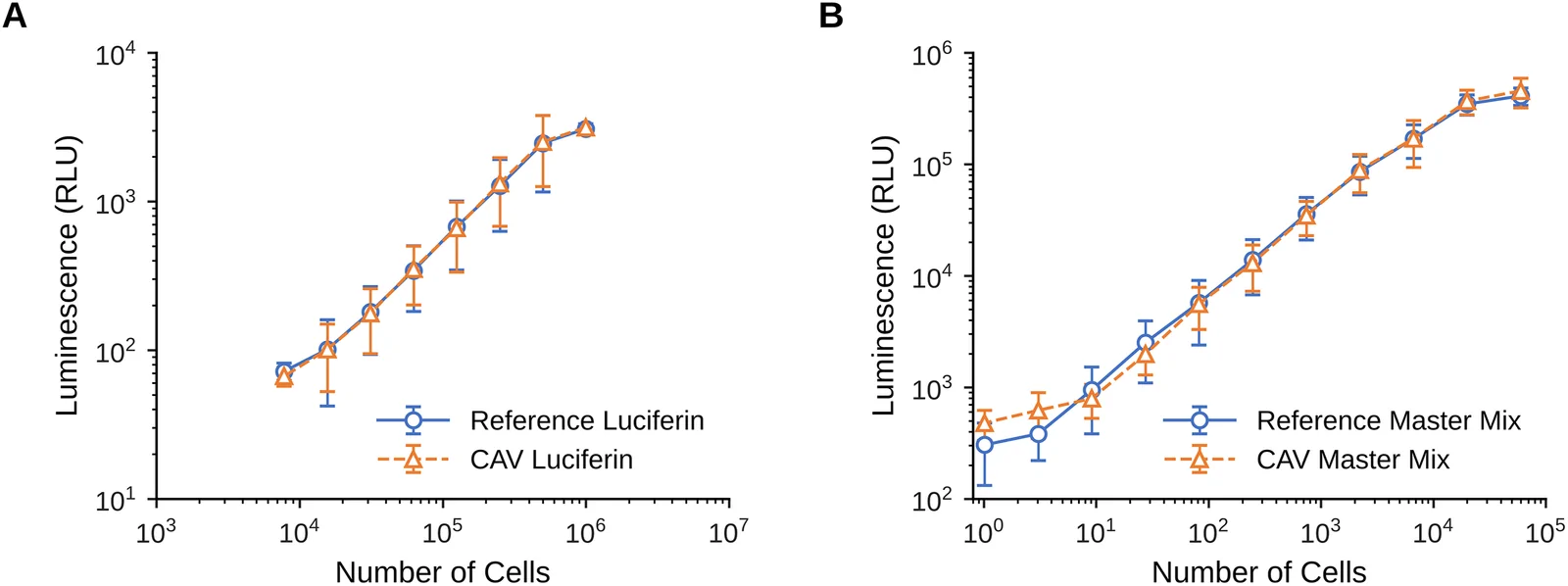
(A) Comparison of the signals generated using reference (blue circles) or CAV-stabilized luciferin (orange triangles) for quantification of gene expression in a 4T1 cell suspension culture (n = 3). (**B**) Comparison of 4T1 cell viability data obtained using reference reagent master mix (blue circles) or CAV-stabilized master mix (orange triangles) (n = 4). Error bars represent standard deviation.

#### Co-formulated master mixes for cell viability and microbial contamination.

As described above, many bioluminescent assays require multiple reagents that, under traditional storage paradigms, must be individually stored. On the day of use, each reagent must be located in the cold storage unit, thawed, diluted and co-formulated to produce a master mix. With the goal of developing thermally stable kits for bioluminescence testing, we prepared stabilized, assay master mixes by co-formulating all of the reagents required to run a specific test method prior to stabilization. On the day of the test, a single scaffold was removed from the packaging, eluted, and immediately used in the appropriate assay. No further reagent preparation or dilution steps were required prior to use. For each experiment, reagents stored in the traditional manner (lyophilized or frozen, with each reaction component stored in a separate vial as shown in Fig 1), were used to prepare a control reagent mixture.

To test the performance of the CAV-stabilized master mix for cell viability applications, freshly cultured 4T1 cells were placed in a 96-well plate and diluted to a range of concentrations ranging from 1 to 6 X 10^4^ cells/well and treated with the reagent master mix. The results obtained with the frozen and CAV-stabilized mixture are visually indistinguishable and not statistically different based on an ANCOVA (Fig 4B). The assay LOQ is estimated to be approximately 13 cells/well using the control master mix and 15 cells/well using the CAV-stabilized master mix.

Given the linear response and sensitivity observed using the 4T1 cells, we evaluated a microbial contamination application. To test for surface contamination (Fig 5), slides were prepared by applying a buffer control (negative condition) or freshly cultured *E. coli*  (1x10^5^ cells for the positive condition). Swabs from the slides were placed into a tube containing either the traditional or CAV-stabilized reagent master mix. The slide was considered positive if the measured signal was above the detection limit. All samples were correctly identified as positive or negative using both the traditional and CAV-stabilized master mix (S1 Table). Although this data successfully demonstrates proof-of-concept, additional testing would be required to verify the limit of detection and confirm comparable sensitivity.

**Fig 5:**
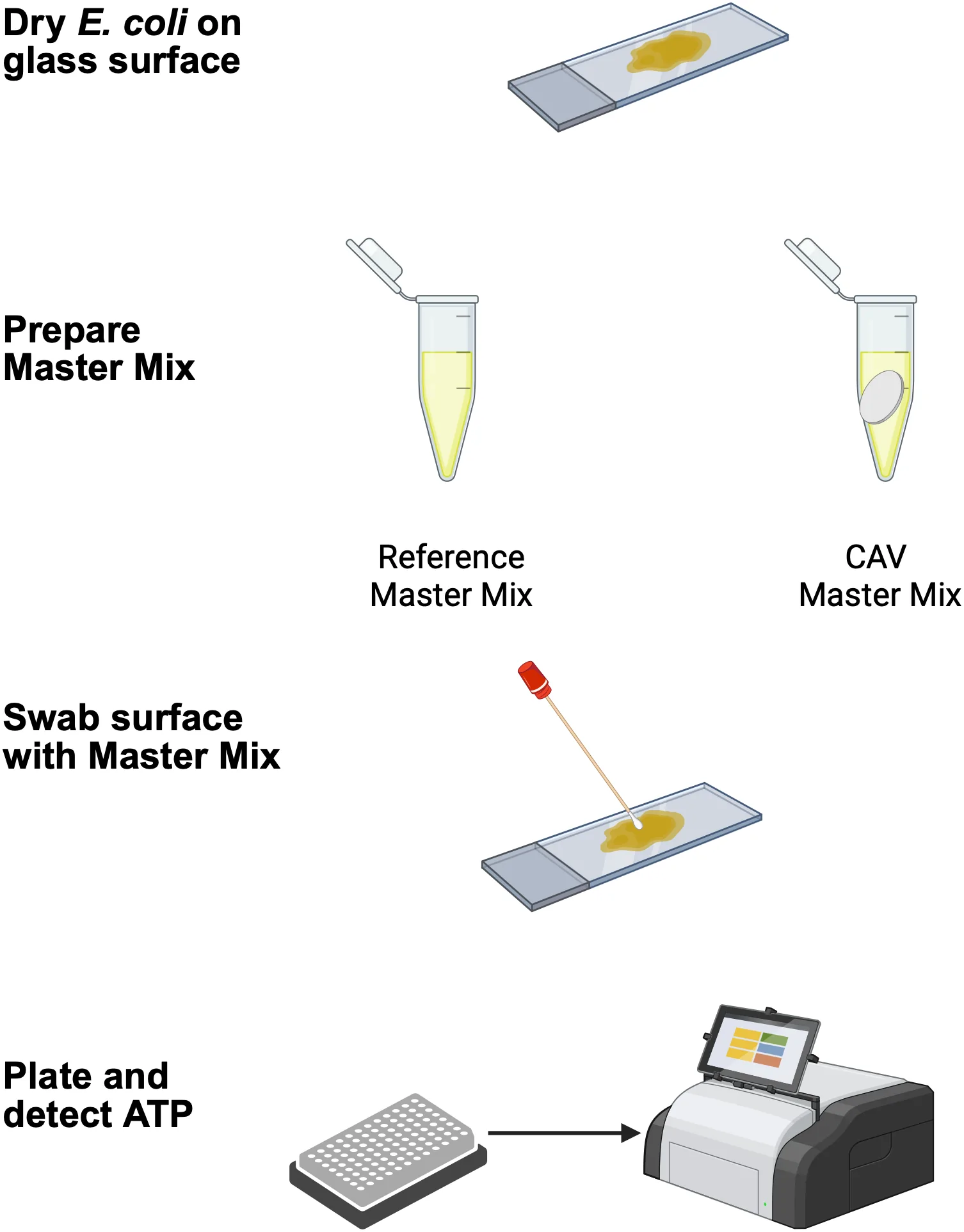
Schematic diagram of the microbial contamination assay. Glass slides were treated with *E. coli* or sterile buffer and dried. The slides were swabbed, and the swab was placed into a tube containing the reaction mixture prepared fresh or from CAV-stabilized reagents. The resulting luminescence was analyzed using a plate reader. Reprinted from Bronsart, L. (2026) https://BioRender.com/x47p386 under a CC BY license, with permission from Biordender original copyright 2026.

#### Homogeneous immunoassay kits.

The Lumit® Flex technology is a homogeneous immunoassay that utilizes a ternary split NanoLuc® luciferase complementation system [33–35]. Two small peptide tags (Peptide α and Peptide β) are chemically or genetically attached to analyte-specific antibodies or binding proteins. A detection polypeptide is then added, and remains inactive until it is brought into proximity to the peptide tags. When the ternary components assemble upon analyte binding, the fully reconstituted NanoLuc enzyme catalyzes oxidation of the furimazine substrate, generating a bright, stable bioluminescent signal. The assay formats show a dose-dependent increase in luminescent signal in response to increasing concentrations of recombinant human IL-6 (rhL-6). While each of the individual reaction components can typically be stored frozen or lyophilized, co-formulation of the components into a ready-to-use mixture can be challenging due to solubility and stability issues, particularly with the furimazine substrate.

Because the CAV-technology has been successfully used to stabilize both small and large molecule reagents, we explored whether CAV stabilization could be used to prepare an interleukin-6 (IL-6) Lumit Flex master mix (Fig 6A). The performance of the standard solution-based Lumit Flex IL-6 immunoassay was compared to CAV-stabilized furimazine alone, a mixture of the two peptide tags and the detection polypeptide, or a master mix in which all of the assay components were co-stabilized. On the day of use, the CAV-reagents were reconstituted in PBS and an rhIL-6 standard curve generated and compared to a standard curve generated with the standard, solution-based reagents. For the furimazine and peptide/polypeptide mixtures, the data was analyzed graphically by plotting the signal-to-noise ratio as a function of rhIL-6 concentration. Using the standard stabilization process with no optimization, the liquid and CAV-stabilized furimazine produced similar signal-to-noise ratios across the range of IL-6 concentrations tested. This suggests that the vitrification did not adversely impact the activity of the substrate or the signal generation process (Fig 6B). Stabilization of the peptide/polypeptide mixture did result in a decrease in signal-to-noise at low IL-6 concentrations relative to the control preparation (Fig 6C). Testing on real-world samples would be required to fully evaluate the impact of the signal shift on the assay sensitivity. However, for most immunoassays, a signal/noise ratio of 10 or more is preferred to ensure adequate precision and accuracy. Using the liquid preparation, IL-6 concentration ranging from 0.07-40 ng/mL met this criterion. For the CAV-stabilized mixture, the range of IL-6 concentrations that yielded an acceptable signal/noise ratio shifted upwards to 0.4-250 ng/mL. The shift in assay response and the reduced signal intensity persisted when all of the assay components including the substrate, peptide tags, and polypeptide were co-formulated and stabilized (Fig 6D).

**Fig 6:**
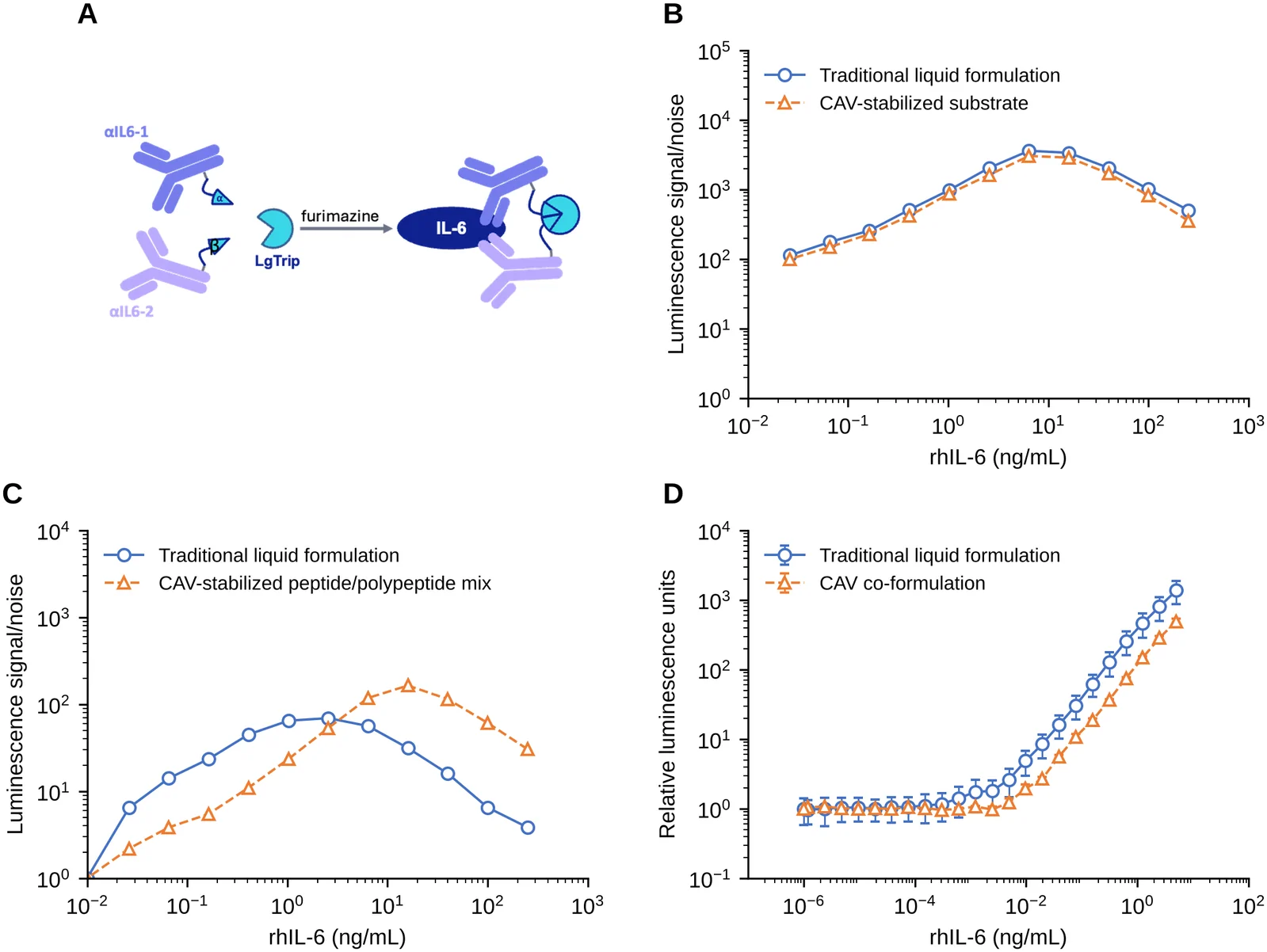
Evaluation of a stabilized Lumit® kit. (A) Schematic diagram of the Lumit® Flex IL-6 Immunoassay. (**B**) Comparison of the signal/noise ratio observed using a traditional liquid preparation of furimazine versus a CAV-stabilized preparation. (**C**) Comparison of the signal/noise ratio observed using a liquid mixture of the two peptide tags and the large polypeptide compared to a CAV-stabilized mixture of the three components. (**D**) Comparison of the performance of Lumit Flex immunoassay using traditional reagent formulation (blue circles) and a co-formulated master mix containing all assay components and stabilized using the CAV process (orange triangles). The CAV stabilized material shows a slight loss in absolute signal and a reduction in sensitivity but good linearity and a broad dynamic range. Error bars indicate the maximum and minimum signals observed across duplicate runs.

Although the solution-based assay exhibits higher absolute signal intensity, the performance of the CAV-stabilized immunoassay may be sufficient for many applications, including studying multiple pathologies, response to therapy, and inflammation [36–38]. Additionally, this data demonstrates the performance that can be achieved with no optimization of either the stabilization protocol or the assay conditions. With optimization, it may be feasible to enhance the sensitivity further

### Scanning electron microscopy

For characterization purposes, scanning electron microscopy (SEM) images were collected of the scaffold material both prior to application of the formulated reagent and after vitrification. For this experiment, the BioFix buffer was used alone. Due to the low concentration of the reagents used in these experiments, it is unlikely that their presence would impact the appearance of the scaffold post-vitrification. Representative images are shown in S3 Fig. As expected, the scaffold has a fibrous appearance with voids, or pores, visible between the fibers (dark regions of the image). After vitrification, a hazy film or glass is visible. The glass appears to coat fibers, resulting in an open, porous structure. This structure is critical for ensuring rapid dissolution of the glass upon rehydration.

## Discussion

Bioluminescence reagents are highly valued for their exceptional sensitivity and robust signal generation and have been successfully implemented across a broad range of analytical applications. Despite their widespread use, thermostability limitations and current storage paradigms continue to present logistical challenges that can hinder successful deployment. Today, most bioluminescent reagents continue to require ultracold storage conditions. This adds significant operational costs and risks loss of valuable materials should the freezer fail. Additionally, the use of frozen reagents adds complexity to the day-to-day execution of the analytical methods; each time an assay is executed, multiple materials must be located, thawed, and diluted, which introduces the opportunity for error and run-to-run variability.

To address these challenges, researchers have explored various stabilization approaches. While genetic engineering may provide limited improvements in luciferase thermostability [16], it is not a solution for small molecule substrates. If carefully optimized and executed, lyophilization and lyobead technologies can in some cases enhance reagent stability as previously noted [22]. However, both methods expose biomolecules to multiple types of stress [24,25] and they are rarely readily available to the bench scientist. Like lyophilization, vacuum foam drying requires complex capital equipment and is not suitable for single-use volumes [39]. While readily accessible, conventional air drying often requires elevated temperatures that can thermally inactivate sensitive enzymes like luciferase and the process is difficult to scale [39].

Our findings demonstrate that CAV offers a distinct and complementary platform. By eliminating the freezing step, the CAV process reduces the risk of cold damage and solubility concerns, allowing researchers to stabilize diverse bioluminescent reagents, both individually and in complex mixtures. The process can be performed with small volumes, making it amenable to storage in single-use quantities. And, as demonstrated here, a single, standardized process can be applied to both large and small molecule reagents with little or no optimization, allowing researchers to easily produce custom assay kits.

The CAV process relies on a unique physical process and the synergistic relationship between the stabilization matrix and the scaffold. The CAV process utilizes a carefully optimized, proprietary mixture of excipients, which like most lyophilization formulations, includes a saccharide component. The saccharide promotes the formation of an amorphous glass matrix, replaces the hydrogen bonds lost when water is removed (water replacement), and furhter enhances stability through preferential exclusion [40].

Unlike lyophilization, where the goal is to produce a freestanding porous glass cake [41], the glass that forms during the CAV process is inherently porous, due to the architecture of the scaffold. Because we utilize the physical architecture of the scaffold to dictate the macroscopic porosity of the final glass, we do not need to rely on the complex physics of freezing to engineer a freestanding porous structure. This reliance on the scaffold’s inherent architecture is a primary reason why CAV requires significantly less formulation optimization compared to traditional lyophilization. Ultimately, this scaffold-driven porosity ensures rapid dissolution of the glass upon rehydration and eliminates the challenges associated with preventing lyo cake collapse.

The choice of the scaffold material is an important consideration as in some cases, there may be an interaction between the material being stabilized and the scaffold material of construction. The most common interaction observed to date is non-specific adsorption or weak binding due to charge-charge and/or hydrophobic interactions between the scaffolds and the biomolecule. In those cases, the issue can generally be resolved by utilizing alternative scaffold materials or modifying the formulation with the addition of surfactants or salts. Scaffolds can be produced from a variety of natural and synthetic polymers provided they offer high porosity and hydrophilicity. The pore size of the materials may vary but attention must be paid to the resultant capillary forces such that liquid absorption capacity is not negatively impacted.

Despite its advantages, the CAV platform has potential molecule- and formulation-specific limitations. While most materials can be stabilized using the standard stabilization protocols, some reagents or assays may require further optimization. In this study, the Lumit Flex immunoassay exhibited a reduction in absolute signal intensity and assay sensitivity compared to the traditional liquid format when stabilized using the standard stabilization protocol. Additionally, although the process is compatible with a broad range of detergents, buffers, salts, chelating agents and other common assay co-factors, the process is not compatible with all excipients. For example, the process can routinely tolerate up to 1% glycerol in the final formulated material, meaning some commercial reagents may require buffer exchange. Finally, the physical characteristics of the scaffold dictate processing limits; each scaffold has a maximum loading volume and a minimum residual hold-up volume, necessitating careful matching of the scaffold size to specific assay requirements to minimize mass loss.

Ultimately, the CAV process offers distinct operational advantages by decoupling reagent stabilization from cumbersome and expensive infrastructure, such as heavy-duty compressors or liquid nitrogen. The compact Ambient Stabilizer can be seamlessly integrated into standard laboratory environments or biological safety cabinets, giving researchers the ability to prepare custom, shelf-stable materials on demand. Eliminating the need to store and ship reagents using cold or ultra-cold storage can have an immediate impact on laboratory carbon footprints and improve global access to research, clinical products, and testing supplies [42]. The thermally stable microbial contamination kit and Lumit Flex immunoassay demonstrate robust analyte detection in a simplified format, highlighting the potential of CAV stabilization to facilitate rapid, on-site testing in decentralized settings such as rural clinics, field hospitals, and outbreak monitoring sites.

## Materials and methods

### Sample preparation

QuantiLum® Recombinant Luciferase, Beetle Luciferin, VivoGlo® Luciferin, and Lumit® Flex human IL-6 assay reagents were obtained from Promega Corp. (Madison, WI). Upon receipt, the luciferase was stored at −80 °C as per the manufacturer’s recommendations. The lyophilized beetle luciferin was reconstituted at 10 mg/mL in phosphate buffered saline (PBS, pH 7.4; Cytiva, Logan UT), aliquoted and stored at −80 °C. VivoGlo Luciferin was reconstituted in a 1:1 mixture of PBS and BioFix stabilization buffer, and aliquots stored at −20 °C.

CAV stabilized samples were prepared in the lab using the Ambient Stabilizer (Ambient Biosciences, Ann Arbor MI). The CAV process was described previously [28], and a schematic of the process is provided in Fig 1. Briefly, the reagent of interest is prepared at a 2X concentration and mixed with an equal volume of BioFix or BioFix+ stabilization buffer (Ambient Biosciences, Ann Arbor MI). The formulated material is dispensed onto a porous material referred to as the scaffold. The quantity of reagent is determined based on the assay requirements and generally each scaffold is designed to hold enough reagent for a single assay run.

The BioFix and BioFix+ buffers are commercially available, preformulated stabilization reagents. The BioFix+buffer contains the same base stabilizers with the addition of a carrier protein which prevents non-specific binding of proteins to surfaces. The BioFix+ is recommended when working with low concentration protein reagents but can be used for other reagents including small molecules.

Scaffolds were selected from a catalog of commercially available options that differ primarily in terms of the volume of reagent that can be applied and stabilized on each scaffold. For each experiment, the smallest scaffold that would hold a suitable volume of the stock reagent was selected. The VivoGlo luciferin, luciferase, and microbial contamination kits were therefore stabilized using BioFix 175 Scaffolds (Ambient Biosciences, Ann Arbor, MI). All other materials were stabilized using BioFix Classic Scaffolds (Ambient Biosciences, Ann Arbor, MI).

On the day of use, the CAV-stabilized samples were rehydrated by incubating the CAV-stabilized samples in elution buffer. The choice of elution buffer was determined separately for each assay based on the assay requirements. Incubation times of 1 minute or less were sufficient to fully rehydrate the CAV-stabilized material. Unless otherwise noted, the eluted material was used in the assay with no further manipulation. Fig 1 provides a schematic of the rehydration process and for comparison purposes, the workflow used to prepare liquid controls.

### Luciferase and luciferin activity assay

Luciferase and luciferin activity were measured using a microtiter plate-based assay. A luciferin reaction mixture containing 1.5 mM beetle luciferin, 4.5 mM magnesium sulfate (Sigma, St. Louis, MO), and 0.5 mM ATP (Sigma, St. Louis MO) was prepared in 25 mM EPPS buffer (Sigma.,St. Louis MO), pH 7.8. Separately, serial dilutions of luciferase ranging in concentration from 0 to 23 µg/mL were prepared. To initiate the assay, a 50 µL aliquot of each luciferase dilution was placed in a 96-well flat-bottom plate with a clear bottom and black walls (Corning, Corning NY) and mixed with a 50 µL aliquot of the luciferin reaction mixture. The plates were incubated for 8 minutes after the addition of all reagents and luminescence was measured using a Synergy H1 microplate reader (BioTek, Winooski, VT) and the endpoint luminescence protocol.

The activity of the test articles was determined by comparing the response of the test article with a reference standard. The reference standard was made using a luciferin mixture and luciferase dilutions that were freshly prepared from frozen stocks on the day of use. The relative activity of the test article was calculated by direct interpolation for each sample dilution that fell within the linear range of the assay and averaging across the dilution adjusted results.

The test articles consisted of one of the following: 1) CAV-stabilized luciferase, 2) luciferase stored in a liquid formulation, 3) CAV-stabilized luciferin, or 4) luciferin stored in a liquid formulation. When luciferase was tested in either the CAV-stabilized or liquid formulation, freshly thawed and diluted luciferin was used. Likewise, when testing luciferin test articles, a freshly prepared luciferase solution was used.

The response curves generated using CAV-stabilized and control reagents were evaluated using a two-side t-test. The Limit of Quantification (LOQ) was determined based on the standard deviation of the response and the slope of the calibration curve. The standard error of the y-intercept generated from the linear regression analysis was utilized as the standard deviation of the response (σ). The LOQ was calculated using the equations LOQ=10σ/S, where S is the slope of the calibration curve.

### Stability assessments

To evaluate the stability of CAV-stabilized luciferase and luciferin, the reagents were initially stabilized individually. CAV-stabilized luciferin samples were prepared so that each scaffold contained 239 µg luciferin in BioFix+ stabilization buffer. At the time of testing, the luciferin scaffolds were eluted with 0.5 mL of a solution containing 4.5 mM magnesium sulfate, 0.5 mM ATP in 25 mM EPPS buffer, pH 7.8. A liquid control was also prepared. The composition of the liquid controls was matched to the CAV-stabilized samples post elution.

CAV-stabilized luciferase samples were prepared so that each scaffold contained 92 µg of luciferase in BioFix+ stabilization buffer. At the time of the test, the luciferase scaffolds were eluted with 4 mL of PBS, producing a solution containing 23 µg/mL luciferase which was then used to prepare serial dilutions for testing. Again, a liquid control was prepared that matched the composition of the luciferase eluted from the BioFix scaffolds, but did not include the BioFix+ stabilization buffer.

The CAV-stabilized samples and liquid controls were stored on the bench (room temperature condition) or in a temperature-controlled incubator set at 55 °C. For the CAV-stabilized samples, three scaffolds were removed from storage at each time point. Triplicate scaffolds were eluted and independently tested, generating three test results per time point. For the liquid controls, a single aliquot was removed at each time point. The liquid control was sampled and tested three times.

### Tumor imaging in mice

All animal experiments were carried out according to a protocol approved by the Vanderbilt University Institutional Animal Care and Use Committee and adhered to all relevant regulations and guidelines. Bioluminescence was evaluated in mice using luciferase-expressing 4T1 cell lines. The CAV-stabilized luciferin samples used in this experiment contained 9 mg of VivoGlo® Luciferin per scaffold. A reference solution was prepared using lyophilized VivoGlo® luciferin reconstituted in PBS. Materials for the tumor imaging studies were shipped to the testing site ambiently via ground transportation and stored at room temperature until use. Ground transportation required 6 days, and during this time the material was exposed to temperatures ranging from 71-107 °F (21–42 °C). The corresponding liquid controls were shipped on dry ice.

Seven-week-old BALB/c female mice (Charles River Laboratories) were implanted with 5 × 10^4^ 4T1-luciferase-expressing cells, suspended in 50 μL of PBS, in the #4 right mammary gland. On the day of use, scaffolds were eluted in 1 mL per scaffold resulting in a final post-elution concentration of 9 mg/mL. Excess reference solution was stored as frozen aliquots between imaging sessions. Bioluminescence imaging 2x/week followed 30 min after a 150 mg/kg i.p. injection of CAV-stabilized luciferin or reference luciferin (n = 7 reference, n = 8 CAV-stabilized). To minimize pain and distress, discomfort produced from tumor implantation was controlled using inhaled isoflurane anesthetic by nose cone (2% isoflurane in 100% O_2_, flow rate 1 L/min). During imaging, each subject was anesthetized on a heated platform using isoflurane delivered via an induction chamber and 2% isoflurane lines. Imaging is non-invasive, and anesthesia was introduced only to keep the subject still during the experiments. We monitored all mice for potential signs of distress over the course of the experiment. Tumors were imaged using an IVIS Lumina Series III imaging system (Caliper LifeSciences). Tumor length and width were measured using digital calipers 2x/week up to 21 days post-inoculation or when humane endpoint (tumor volume>1,000 mm^3^) was reached. Tumor volume was calculated using the formula: Volume = (D_1_^2^ x D_2_)/2, where D_1_ is the minimum diameter and D_2_ is the maximum diameter. Tumors reached an average of 650 ± 238 mm^3^ (mean ± SD) and one mouse died before meeting the criteria for euthanasia.

### 4T1 reporter gene expression

A luciferase-expressing mouse mammary carcinoma cell line 4T1/Puro Dual Labeled Stable Cell Line was obtained from GeneCopoeia (SL020). The cell line is stably transfected with both a firefly luciferase gene and eGFP gene. Cells were propagated and maintained in RPMI selection medium (BioWhittaker/Lonza, Walkersville MD and Gibco, Grand Island NY) containing 1% GlutaMAX (Gibco, Grand Island NY), 1% Ab/Am, puromycin (Gibco, Grand Island NY) and 10% FBS (Gibco, Grand Island NY). Cells were cultured at 37 °C in a 5% CO_2_ atmosphere.

On the day of testing, cells were suspended in medium containing 450 µg/mL luciferin and titrated from 7.8 X 10^3^ to 10^6^ cells per well in 2-fold serial dilutions in an opaque white 96-well cell culture plate (Corning, Corning NY). Prior to use, the luciferin was either stored frozen and thawed and diluted in media on the day-of-use (reference solutions) or in the CAV stabilized form. Each CAV-stabilized aliquot contained 2.7 mg of beetle luciferin and was eluted in 6 mL of media. After a 15-minute exposure, bioluminescence intensity was read using a Synergy H1 microplate reader. The experiment was carried out on each of 3 days for n = 3 replicate runs. The relative activity for the CAV-stabilized luciferin was determined by calculating the average of all dilutions that fell within the linear range of the assay and reporting that result as a percentage relative to the reference standard. ANCOVA analysis was performed to test for statistical differences in the data set. The limit of quantitation (LOQ) was defined as the mean blank signal plus 10 times the standard deviation of the blanks, respectively. These signal thresholds were subsequently converted to concentrations by interpolation against the log-log linear regression equation of the raw, uncorrected standard curve.

### Cell viability assessment

Bioluminescence was evaluated in CRL-2539 4T1 cells (ATCC, Manassas VA) to evaluate whether luciferase and luciferin could be co-formulated into an easy-to-use assay master mix or kit for cell viability testing. This cell line does not contain a luciferase reporter gene. Cells were titrated from 1 to 6 × 10^4^ cells/well in an opaque, white 96-well cell culture plate, centrifuged, and washed with Dulbecco’s Phosphate Buffered Saline (DPBS, Lonza, Walkersville MD). A 20 µL aliquot of lysis buffer containing 25 mM tris-phosphate (pH 7.8), 2 mM dithiothreitol, 2 mM EDTA, 10% glycerol, 1% Triton X-100 was added to each well and the plate was incubated on a shaker for 20 minutes. Scaffolds containing 4.4 µg luciferase and 840 µg luciferin co-vitrified in BioFix+ stabilization buffer were prepared. Six scaffolds were eluted together in 5.28 mL buffer containing 4.5 mM magnesium sulfate and 25 mM EPPS, pH 7.8, for a final concentration of 5 µg/mL luciferase and 3 mM luciferin. A reference standard was prepared from freshly thawed luciferase and luciferin diluted to 5 µg/mL luciferase and 3 mM luciferin in buffer containing 4.5 mM magnesium sulfate, 0.28% bovine serum albumin (BSA), 25 mM EPPS, pH 7.8. Subsequently, plate wells were filled with 100 µL master mix with sample or standard. After a 15-minute exposure, bioluminescence intensity was read using a Synergy H1 microplate reader. The relative activity for the CAV-stabilized master mix was determined by calculating the average across all dilutions that fell within the linear range of the assay and reporting that result as a percentage relative to the reference standard. ANCOVA analysis was performed to test for statistical differences in the data set. Using only data that fell within the linear range of the assay (10–2222 cells/well) the LOQ was defined as the mean blank signal plus 10 times the standard deviation of the blanks. These signals were subsequently converted to concentrations by interpolation against the log-log linear regression equation of the standard curve.

### Microbial contamination testing

A microbial contamination kit was prepared by vitrifying 0.875 µg luciferase, 139 µg luciferin, 9 µg DTT, and 0.035 µg Triton X-100 in BioFix+ stabilization buffer on BioFix 175 scaffolds. This master mix contained the enzyme, substrate and all co-factors required for testing in a single CAV-stabilized preparation. Sterile broth or broth containing 1 X 10^5^ *E. coli* cells were applied to sterile microscope slides and allowed to dry for 2 hours at room temperature. On the day of the test, a liquid reference control mixture was prepared from 4.16 µg/mL luciferase, 2.5 mM luciferin, 0.333 mM DTT, 2% glycerol, 0.2% Triton X-100, 3.5 mM MgSO_4_ in 25 mM Tris buffer. CAV scaffolds were eluted in 0.5 mL buffer containing 2% glycerol, 3.5 mM MgSO_4_ in 25 mM Tris. A FLOQSwab (Copan Cat #534CS01) was soaked in 0.5 mL reaction mixture, used to separately swab the surface of each glass slide, and then returned to a tube containing the reference master mix prepared from frozen stocks or from the CAV-stabilized master mix. A 100 µL quantity of the reaction mixture was then transferred to a 96-well plate and read for luminescence in a Synergy H1 plate reader. Limit of detection was defined as the mean of the negative control plus three times the standard deviation of those six negative control samples. In this dataset, LOD was defined as 798 RLU. Anything above this threshold was considered positive. The microbial contamination assay format is shown schematically in Fig 5.

### Lumit® Flex IL-6 immunoassay

Anti-IL-6 antibody clones 5IL-6 and 505E (Thermo Fisher) were chemically conjugated with the Lumit® Flex alpha and beta peptide (Promega Corp), respectively, and optimized for working concentration as previously reported [33]. Briefly, each antibody was incubated with an amine-reactive form of the peptide labels for 1 hour at room temperature, quenched with 2 M Tris buffer, and then diluted in 80% glycerol for storage. A solution-based assay was established using a master mix containing 2 µM Lumit® Flex Detection Protein, 5IL-6-Lumit® Flex Peptide Alpha labeled antibody, 505E-Lumit® Flex Peptide Beta labeled antibody, and 10 µM of Nano-Glo Live Cell Substrate (Promega Corp). This master mix/coformulation was mixed with recombinant IL-6 (R&D Systems) titration prepared in assay buffer. After 1.5 h, the luminescence was read on a GloMax Discover Luminometer (Promega Corp).

The CAV-stabilization of the Lumit Flex IL-6 immunoassay was performed using the BioFix+ buffer. Scaffolds were prepared containing the substrate only, the two labeled peptides plus the polypeptide detection protein, or all of the assay components co-formulated together. Post-stabilization, scaffolds were eluted with 500 µL of PBS + 0.01% BSA to collect the assay components, which were then added to a non-binding white 96-well plate (Corning) containing a dilution of IL-6.

### Scanning Electron Microscopy (SEM)

SEM images of a BioFix scaffold before application of any liquid and post-vitrification were collected without sputter coating using a Thermo Fisher Quattro ESEM in low vacuum mode at a pressure of 0.25 Torr. Images were collected with an accelerating voltage of 10.00 kV, current of 0.37 nA, and a horizontal field width of 300 µm.

## Supporting information

S1 Fig Luciferin stability. Activity of luciferin as a function of time when stored in the CAV-stabilized form either at room temperature (orange triangles) or 55 °C (pink diamonds) or as a liquid at room temperature (blue circles). Data are consistent with a first-order reaction model and are therefore plotted as the natural log of the measured activity versus time. The positive control served as the assay reference and was diluted from 10 to 2.1 µg/mL in triplicate. For each vitrified test article, we eluted 3 scaffolds, and a single dilution series spanning the same concentration range was prepared from each scaffold.
(DOCX)

S2 Fig Luciferase stability. Activity of CAV-stabilized luciferase as a function of time when stored at room temperature (orange triangles) or 55 °C (pink diamonds). Stability trends appear to follow first-order kinetics, and the results are therefore plotted as the natural log of the activity versus time. Data for liquid luciferase formulations not shown. At 55 °C, the luciferase stored as a liquid lost all activity in less than 1 day. At 25 °C, the test results for the liquid luciferase were not reproducible across time points or repeat stability studies. The positive control served as the assay reference and was diluted from 10 to 2.1 µg/mL in triplicate. For each vitrified test article, we eluted 3 scaffolds, and a single dilution series spanning the same concentration range was prepared from each scaffold.
(DOCX)

S1 Table Detection of microbial contamination. Raw signals and reportable results obtained for the microbial detection assay using traditional, liquid reagents versus the CAV-stabilized master mix.
(DOCX)

S3 Fig Representative SEM images of the scaffold material (left) and of a vitrified sample (right). The images show the fibrous nature of the scaffold material with the darker areas representing voids or pores in the material. Post-vitrification, an amorphous glass forms and coats the fibers. This produces a porous glass with high surface area, enabling rapid dissolution.
(DOCX)
