# Supplementary material for: Development of shelf-stable reagents and assay kits for bioluminescence applications using the capillary-assisted vitrification platform stabilization technology
Source: PLoS One. 2026 Nov 5;21(11):e0354533. doi: 10.1371/journal.pone.0354533 (PMC13739107; doi:10.1371/journal.pone.0354533)
Supplement: Supplementary file 1 [file pone.0354533.s001.docx]

**S1 Fig. Luciferin Stability.** Activity of luciferin as a function of time when stored in the CAV-stabilized form either at room temperature (orange triangles) or 55 °C (pink diamonds) or as a liquid at room temperature (blue circles). Data are consistent with a first-order reaction model and are therefore plotted as the natural log of the measured activity versus time. The positive control served as the assay reference and was diluted from 10-2.1 mcg/mL in triplicate.  For each vitrified test article, we eluted 3 scaffolds, and a single dilution series spanning the same concentration range was prepared from each scaffold.
