# Supplementary material for: Development of shelf-stable reagents and assay kits for bioluminescence applications using the capillary-assisted vitrification platform stabilization technology
Source: PLoS One. 2026 Nov 5;21(11):e0354533. doi: 10.1371/journal.pone.0354533 (PMC13739107; doi:10.1371/journal.pone.0354533)
Supplement: Supplementary file 2 [file pone.0354533.s002.docx]

**S1 Fig. Luciferin Stability.** Activity of luciferin as a function of time when stored in the CAV-stabilized form either at room temperature (orange triangles) or 55 °C (pink diamonds) or as a liquid at room temperature (blue circles). Data are consistent with a first-order reaction model and are therefore plotted as the natural log of the measured activity versus time. The positive control served as the assay reference and was diluted from 10-2.1 mcg/mL in triplicate.  For each vitrified test article, we eluted 3 scaffolds, and a single dilution series spanning the same concentration range was prepared from each scaffold.

**S2 Fig.** **Luciferase stability.** Activity of CAV-stabilized luciferase as a function of time when stored at room temperature (orange triangles) or 55 °C (pink diamonds).  Stability trends appear to follow first-order kinetics and the results are therefore plotted as the natural log of the activity versus time. Data for liquid luciferase formulations not shown. At 55°C, the luciferase stored as a liquid lost all activity in less than 1 day. At 25°C, the test results for the liquid luciferase were not reproducible across time points or repeat stability studies. The positive control served as the assay reference and was diluted from 10-2.1 mcg/mL in triplicate.  For each vitrified test article, we eluted 3 scaffolds, and a single dilution series spanning the same concentration range was prepared from each scaffold.
