# Supplementary material for: Development of shelf-stable reagents and assay kits for bioluminescence applications using the capillary-assisted vitrification platform stabilization technology
Source: PLoS One. 2026 Nov 5;21(11):e0354533. doi: 10.1371/journal.pone.0354533 (PMC13739107; doi:10.1371/journal.pone.0354533)
Supplement: Supplementary file 4 [file pone.0354533.s004.docx]

**S3 Fig. Representative SEM images of the scaffold material (left) and a of a vitrified sample.** The images show the fibrous nature of the scaffold material with the darker areas representing voids or pores in the material. Post-vitrification, an amorphous glass forms and coats the fibers. This produces a porous glass with high surface area, enabling rapid dissolution.
